# Mechanism of Random Telegraph Noise in 22-nm FDSOI-Based MOSFET at Cryogenic Temperatures

**DOI:** 10.3390/nano12234344

**Published:** 2022-12-06

**Authors:** Yue Ma, Jinshun Bi, Hanbin Wang, Linjie Fan, Biyao Zhao, Lizhi Shen, Mengxin Liu

**Affiliations:** 1 Institute of Microelectronics, Chinese Academy of Sciences, Beijing 100029, China; 2 School of Microelectronics, University of Chinese Academy of Sciences, Beijing 100049, China; 3 Beijing Zhongke New Micro Technology Development Co., Ltd., Beijing 100029, China

**Keywords:** random telegraph noise (RTN), fully depleted silicon-on-insulator (FDSOI), cryogenic temperatures, trap depth, inversion layer thickness

## Abstract

In the emerging process-based transistors, random telegraph noise (RTN) has become a critical reliability problem. However, the conventional method to analyze RTN properties may not be suitable for the advanced silicon-on-insulator (SOI)-based transistors, such as the fully depleted SOI (FDSOI)-based transistors. In this paper, the mechanism of RTN in a 22-nm FDSOI-based metal–oxide–semiconductor field-effect transistor (MOSFET) is discussed, and an improved approach to analyzing the relationship between the RTN time constants, the trap energy, and the trap depth of the device at cryogenic temperatures is proposed. The cryogenic measurements of RTN in a 22-nm FDSOI-based MOSFET were carried out and analyzed using the improved approach. In this approach, the quantum mechanical effects and diffuse scattering of electrons at the oxide–silicon interface are considered, and the slope of the trap potential determined by the gate voltage relation is assumed to decrease proportionally with temperature as a result of the electron distribution inside the top silicon, per the technology computer-aided design (TCAD) simulations. The fitted results of the improved approach have good consistency with the measured curves at cryogenic temperatures from 10 K to 100 K. The fitted trap depth was 0.13 nm, and the decrease in the fitted correction coefficient of the electron distribution proportionally with temperature is consistent with the aforementioned assumption.

## 1. Introduction

With the scaling down and utilization of high-k metal gates (HKMGs) in metal-oxide-semiconductor field-effect transistors (MOSFETs), the occurrence of random telegraph noise (RTN), especially the generation of random telegraph signals (RTSs), is becoming a critical reliability problem in analog integrated circuits (ICs), digital ICs, and the memories due to the shift in threshold voltage from the capture and emission of carriers by traps inside the gate oxide [1,2,3,4,5]. Moreover, RTN is also a severe reliability problem in cryogenic quantum computing applications [6]. The integrated quantum processor, which contains quantum bits (q-bits) and peripheral circuits, operates at cryogenic temperatures. Because its peripheral circuits are based on MOSFETs, the RTN from the cryogenic MOSFETs may cause reliability problems in the integrated quantum processor. Besides, RTN can also cause reliability problems in other applications whose peripheral circuits are based on MOSFETs, such as the two-dimensional material-based applications [7,8,9,10]. Past research studying the mechanism of RTN on cryogenic bulk MOSFETs has already been presented, where primarily, the properties of RTN, such as time constants and trap depth, have been discussed [6,11,12]. However, the mechanism of RTN in the cryogenic fully depleted silicon-on-insulator (FDSOI) MOSFET, especially the relationship between the time constant, trap energy, and trap depth, is hardly mentioned in recent works. Considering that FDSOI MOSFETs are promising candidates for the peripheral circuits in integrated quantum processors, it is necessary to investigate the mechanism of RTN at cryogenic temperatures in FDSOI MOSFETs.

**Figure 1:**
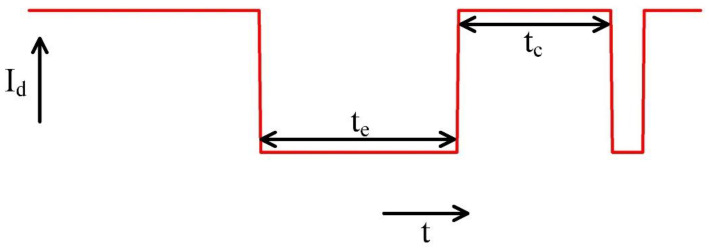
RTN in MOSFET, $t_{c}$ is the capture time and $t_{e}$ is the emission time.

In MOSFETs, both bulk and FDSOI MOSFETs, traps in the gate oxide may capture carriers from the channel or release (emit) carriers into the channel. This capture or emission of carriers can temporarily change the threshold voltage of the MOSFET, leading to a temporary shift in the drain current, as shown in Figure 1. The duration of capture and emission of carriers are denoted by $t_{c}$ and $t_{e}$, respectively.

**Figure 2:**
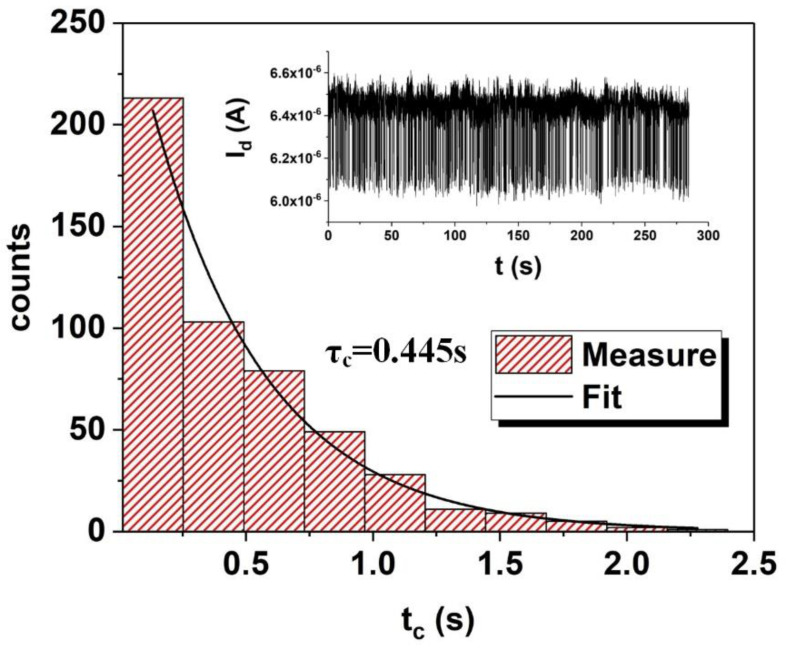
The fitting of $\tau_{\alpha }$ to the Poisson distribution and the extraction of $\tau_{\alpha }$.

The $t_{c}$ and $t_{e}$ appear randomly, obeying the Poisson distribution, and can be described as [13,14]:

(1) $$P_{\alpha }\left(t_{\alpha }\right)=\frac{1}{\tau_{\alpha }}\exp\left(-\frac{t_{\alpha }}{\tau_{\alpha }}\right)\;\;\alpha =c\;or\;e$$

where, $\tau_{\alpha }$ is the time constant of the capture or emission; $P_{\alpha }\left(t_{\alpha }\right)dt_{\alpha }$ is the probability that the capture or emission occurs between $t_{\alpha }$ and $t_{\alpha }+dt_{\alpha }$. To extract $\tau_{\alpha }$, the distribution of $t_{\alpha }$ is fitted to Equation (1), as shown in Figure 2. The measured data are the counts of different $t_{\alpha }$; by fitting them to the Poisson distribution, $\tau_{\alpha }$ can be extracted.

To understand the relationship between the time constants $\tau_{\alpha }$ and the trap energy $E_{T}$, the grand partition function is adopted. According to the grand partition function, the relationship between $\tau_{\alpha }$ and $E_{T}$ can be described by [13,15]:

(2) τc=τ0·[1+e(ET−EF)kT]

(3) τe=τ0·[1+e−(ET−EF)kT]

where, $\tau_{0}$ is the characteristic time constant, $E_{F}$ is the Fermi level, k is the Boltzmann constant, and T is the absolute temperature. Then, it can be inferred that:

(4) $$ln\frac{\tau_{c}}{\tau_{e}}=\frac{\left(E_{T}-E_{F}\right)}{kT}$$

In the bulk MOSFET, when the device is operating in the strong inversion region (assuming the potential of the inversion layer is pinned to the gate voltage), the trap depth can be extracted by Equation (4) [16,17,18,19,20]:

(5) XTtox=−kTqd lnτcτed Vg

where, $X_{T}$ is the trap depth from the oxide-silicon interface, $t_{ox}$ is the thickness of the gate oxide, $V_{g}$ is the gate voltage, and q is the charge of the electron.

Although the bulk MOSFET and the FDSOI MOSFET are both planar devices, it is still uncertain as to whether the relationship between the RTN time constants, the trap energy, and the trap depth in the bulk MOSFET (Equation (5)) is suitable for the FDSOI MOSFET or not. Thus, exploring an appropriate method to analyze the RTN properties in FDSOI MOSFETs, especially at cryogenic temperatures, is meaningful for reliability analysis in emerging SOI processes.

In this paper, the mechanism of RTN at cryogenic temperatures on a 22-nm FDSOI MOSFET is reported, and the relationship between the time constants, trap energy, and trap depth at cryogenic temperatures of the 22-nm FDSOI MOSFET is discussed. The paper is organized as follows: Section 2 introduces the experimental configuration of the cryogenic measurement of RTN in the 22-nm FDSOI MOSFET. Next, Section 3 presents the measurement results and the problems with the analysis of RTN. Then, Section 4 attempts to explain the problems mentioned in Section 3 and proposes an improved approach to analyzing the RTN in FDSOI MOSFETs at cryogenic temperatures. Finally, Section 5 makes a brief conclusion.

## 2. Experimental Configurations

To measure the RTN properties of the 22-nm FDSOI MOSFET at cryogenic temperatures, the device under test (DUT) of this experiment is based on the 22FDX technology from Global Foundries [21,22]. The 22FDX technology provides low threshold voltage (LVT) and super low threshold voltage (SLVT) N- and P-type MOSFETs with different gate lengths (L) and widths (W). For the experiment, a LVT N-type MOSFET with W/L = 160 nm/20 nm was chosen. The main dimensions of the chosen MOSFET are shown in Table 1. In Table 1, t_Si_, t_BOX_, t_OX_, L_g_, and W are the thickness of the top silicon, thickness of the buried oxide (BOX), thickness of the gate oxide, channel length, and channel width, respectively. The 22FDX technology uses the HKMG technology, where the traps inside the high-k gate oxide or at the oxide-silicon interface make the RTN problems worse.

The cryogenic experiments were conducted between 10 K and 100 K on a Lakeshore cryogenic probe station, and the RTN measurements were performed using the Keithley 4200A. To explore the changes in the properties related to RTN, in the measurements, the drain voltage was kept constant while $V_{g}$ was varied and the time-domain characteristics of the drain current were sampled.

## 3. Measurement Results

**Figure 3:**
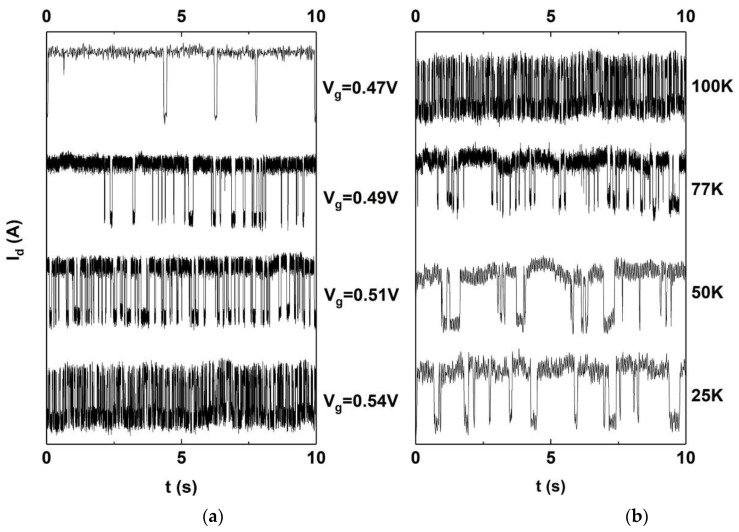
Time-domain graphs of RTN. (**a**) varying $V_{g}$ at 100 K; (**b**) varying T at $V_{g}$ = 0.54 V.

In the cryogenic RTN measurements on the FDSOI, it was observed that the $t_{c}$ (high) and $t_{e}$ (low) vary with the change in the $V_{g}$ and T, which reveals that the time constants also vary with the $V_{g}$ and T, as shown in Figure 3. Thereafter, the time constants were extracted as introduced in Section 1.

**Figure 4:**
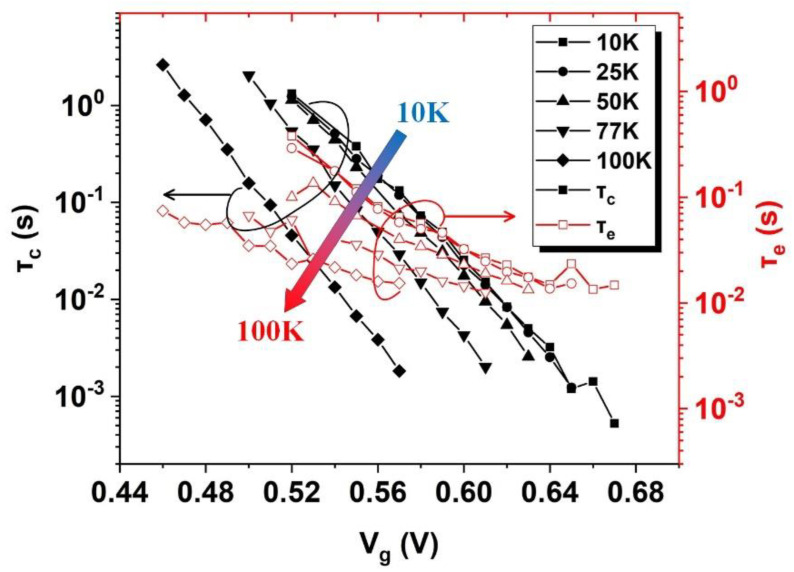
Plots of $\tau_{c}$ (solid) and $\tau_{e}$ (open) versus $V_{g}$ at cryogenic temperatures ranging from 10 K to 100 K.

Figure 4 illustrates the relationship of $\tau_{c}$ and $\tau_{e}$ with $V_{g}$ at different cryogenic temperatures from 10 K to 100 K. With the increase in temperature, both $\tau_{c}$ and $\tau_{e}$ decrease. However, when $V_{g}$ decreases, $\tau_{c}$ increases exponentially, while $\tau_{e}$ only slightly increases. This is different from the phenomenon in [23] where $\tau_{c}$ increases exponentially and $\tau_{e}$ decreases exponentially with the decrease in $V_{g}$ at room temperature.

**Figure 5:**
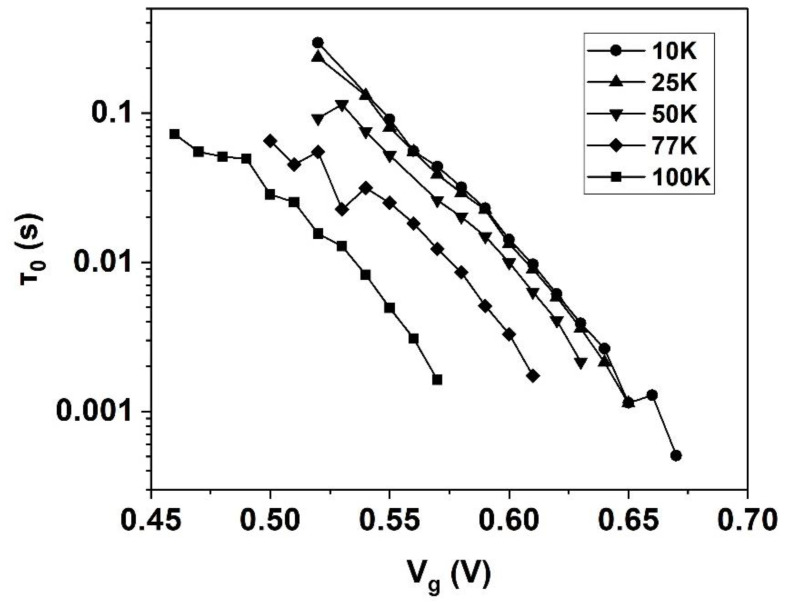
$\tau_{0}$ versus $V_{g}$ at different cryogenic temperatures.

At room temperature, the characteristic time constant, $\tau_{0}$, in Equations (2) and (3) is considered as constant that will not change with $V_{g}$. Thus, the relationship of $\tau_{c}$ and $\tau_{e}$ with $V_{g}$ according to Equations (2) and (3) would be different than what was observed in this study: if one time constant increases exponentially, the other should decrease exponentially [23]. However, according to the thermal activation theory [13], $\tau_{0}$ may not be treated as a constant at cryogenic temperatures. Figure 5 shows the plots of $\tau_{0}$ with $V_{g}$ of the FDSOI at different cryogenic temperatures. It can be seen that $\tau_{0}$ decreases exponentially as $V_{g}$ increases, which may be due to the increasing carrier density in the inversion layer with the increase of $V_{g}$.

**Figure 6:**
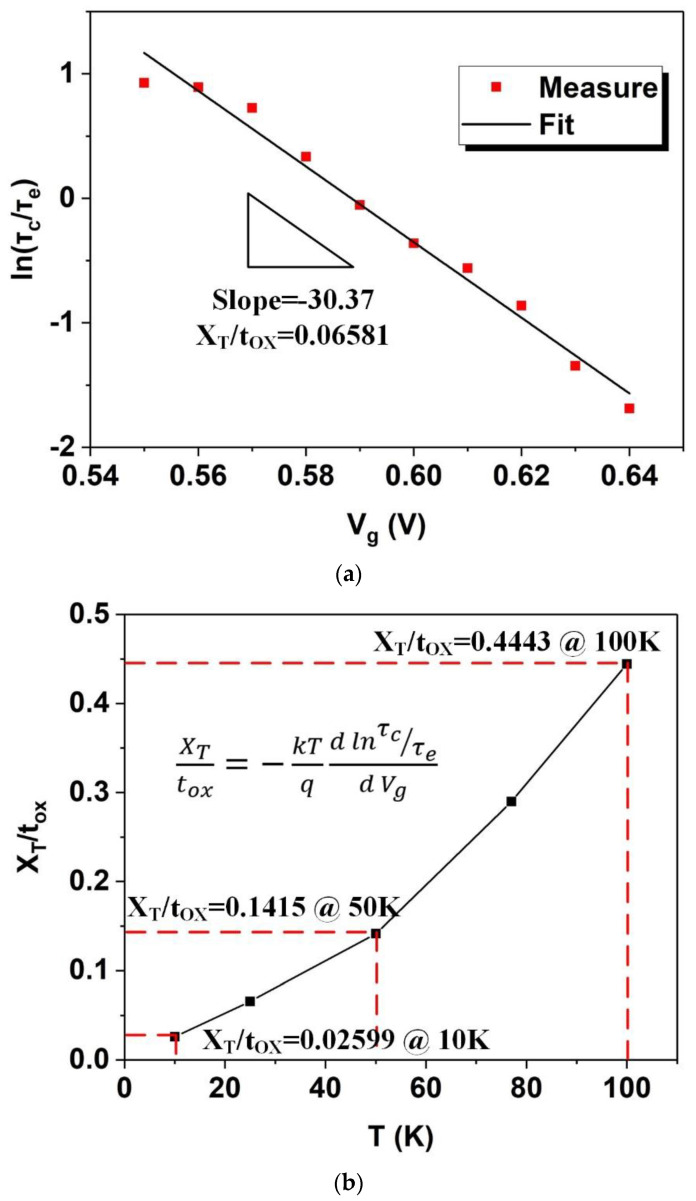
(**a**) Extracting the trap depth at 25 K; the extracted slope is −30.37, and the calculated $\frac{X_{T}}{t_{ox}}$ is 0.06581. (**b**) Plot showing the calculated trap depth versus temperature.

To extract the trap depth using Equation (5), $ln\frac{\tau_{c}}{\tau_{e}}$ is calculated, plotted by $V_{g}$, and linearly fitted to extract the slope. Figure 6a shows the fitting procedure at T of 25 K. The slope of the fitted curve is −30.37, and according to Equation (5), $\frac{X_{T}}{t_{ox}}$ is 0.06581. Similarly, the trap depth was extracted for cryogenic temperatures of 10 K, 50 K, 77 K, and 100 K as well. The absolute errors between measured and linearly fitted $ln\frac{\tau_{c}}{\tau_{e}}$ curves are illustrated in Table 2. The total maximum error and total average error are 0.63789 and 0.13280, respectively. As shown in Figure 6b, $\frac{X_{T}}{t_{ox}}$ falls from 0.4443 at 100 K to 0.02599 at 10 K. This is an abnormal phenomenon because the location of the traps in the gate oxide is unlikely to change with the change in temperature in the FDSOI MOSFET. Moreover, this also implies that the conventional method of extracting the trap depth—i.e., Equation (5)—is probably not suitable for newer types of MOSFETs, such as the FDSOI MOSFET.

## 4. Discussion

In the conventional trap depth extraction, the basic assumptions are that the potential of the inversion layer is constant and the peak of the carrier density is at the oxide-silicon interface, which are suitable for the bulk MOSFET. To extract the trap depth, from Equation (4), the first derivative of $ln\frac{\tau_{c}}{\tau_{e}}$ with respect to $V_{g}$ is taken [24]:

(6) d lnτcτed Vg=−qkTd VTrapd Vg

where, $V_{Trap}$ stands for the trap potential.

**Figure 7:**
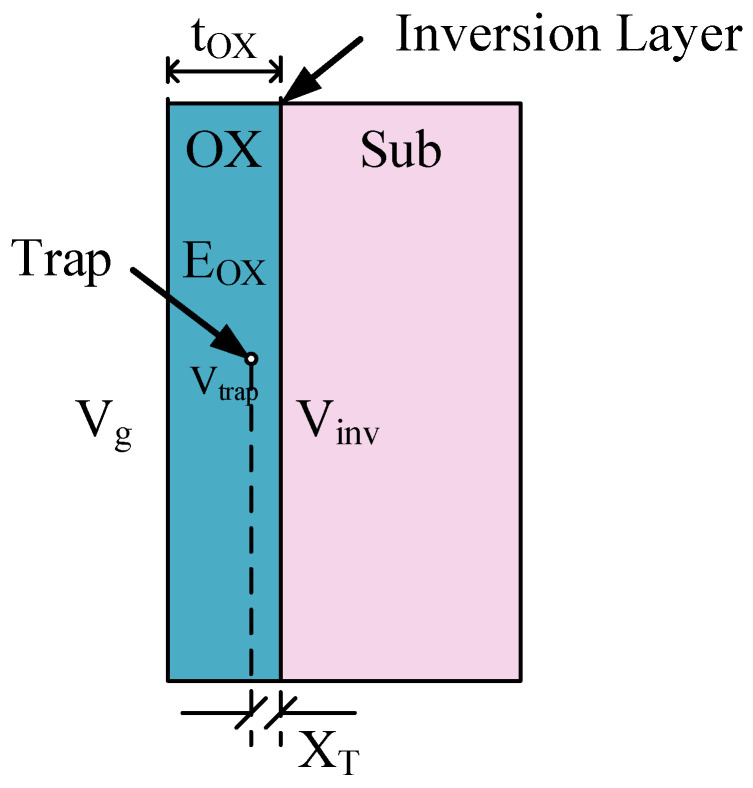
The geometric relationship between $V_{g}$, $V_{Trap}$, and $X_{T}$.

Figure 7 illustrates the geometric relationship between the $V_{g}$, $V_{Trap}$, and $X_{T}$. It can be inferred easily that:

(7) $$\;V_{trap}=V_{inv}+E_{OX}\;X_{T}=\frac{\;V_{g}-V_{inv}}{\;t_{OX}}\;X_{T}+V_{inv}=V_{g}\frac{\;X_{T}}{\;t_{OX}}+V_{inv}\left(1-\frac{\;X_{T}}{\;t_{OX}}\right)$$

where, $V_{inv}$ is the inversion layer potential and $E_{OX}$ is the electrical field intensity inside the gate oxide. Assuming that $V_{inv}$ is constant in the strong inversion region, the first derivative of $V_{trap}$ with respect to $V_{g}$ is:

(8) $$\frac{d\;V_{Trap}}{d\;V_{g}}=\frac{\;X_{T}}{\;t_{OX}}$$

Thus, combining Equations (6) and (8), Equation (5) can be derived.

Nevertheless, these assumptions, while suitable for bulk MOSFETs, may not be precise for FDSOI MOSFETs. In the FDSOI MOSFET, carriers are generated inside the entire top silicon, and most of the time the peak of the carrier density is not located at the oxide-silicon interface due to quantum processes [25,26,27,28,29,30,31] and diffuse scattering [32,33]. Thus, the geometric relationship shown in Figure 7 is not precise for FDSOI MOSFETs, and Equation (8) cannot be derived by the differentiation of $V_{trap}$. Although Equations (5), (7) and (8) are no longer suitable for the FDSOI MOSFET, the slope of $V_{trap}$ to $V_{g}$ is still an important trap-related characteristic.

**Figure 8:**
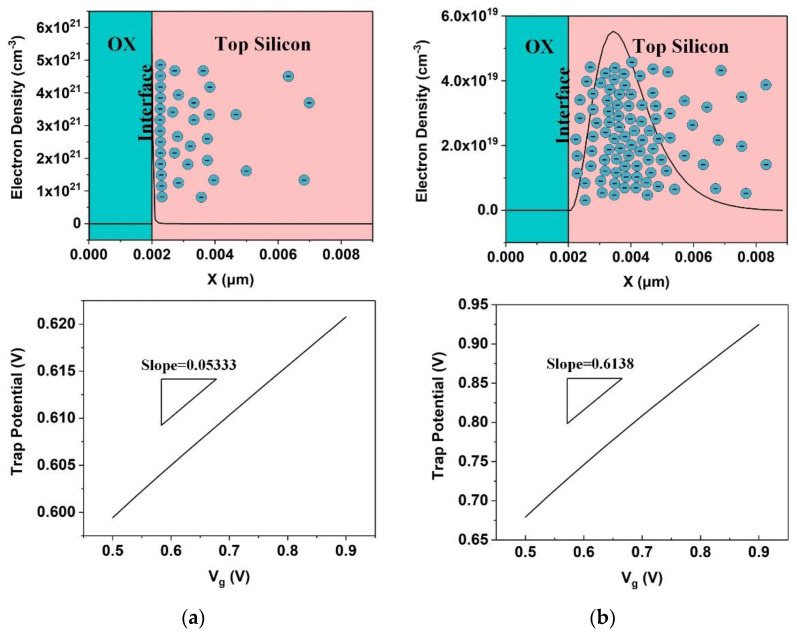
The results of the simulations: (**a**) at 10 K with quantum correction disabled and (**b**) at 100 K with quantum correction enabled.

To explore the influence of the carrier distribution in the top silicon on the slope of $V_{trap}$ with respect to $V_{g}$, TCAD simulations were performed. The TCAD simulations were implemented by Silvaco TCAD tools, and the quantum correction method is a self-consistent coupled Schrodinger-Poisson model. The device structure in the simulations is consistent with the real geometric size of a 22-nm FDSOI MOSFET as shown in Table 1. Figure 8 illustrates the simulated results. The cutline of the electron density is in the middle of the channel, and the trap depth is set to 0.1 nm. Figure 8a, shows the results of the simulation that was performed at 10 K while the quantum correction was disabled. It can be observed that the peak of the electron density is at the oxide-silicon interface, resulting in a $V_{trap}$ − $V_{g}$ slope of 0.05333. The slope from the simulation has good consistency with Equation (8), where $\frac{\;X_{T}}{\;t_{OX}}$ = 0.05. In Figure 8b, the temperature of the simulation was 100 K and the quantum correction was enabled. As a result of the quantum correction and the diffuse scattering, the peak of the electron density is at about 1.5 nm from the interface, and the electrons are distributed in a wide range inside the top silicon. Hence, the slope of $V_{trap}$ − $V_{g}$ is greater than that in Figure 8a: 0.6138. The simulations demonstrate that the slope of $V_{trap}$ with respect to $V_{g}$ is strongly related to the distribution of electrons inside the top silicon and that the non-ideal electron distribution caused by quantum processes and diffuse scattering significantly increases the slope. Therefore, according to Equation (8), non-ideal electron distribution can also lead to a significant increase in the calculated trap depth.

As shown in Figure 6b, the calculated trap depth increases with temperature. Additionally, as discussed before, the non-ideal distribution of electrons increases the calculated trap depth as well. Thus, it can be deduced that temperature has a significant influence on the electron distribution inside the top silicon. To investigate this relationship, the quantum mechanical processes and diffuse scattering are first considered. Previous studies have already proven that the inversion layer thickness—the average distance from the oxide-silicon interface to electrons—is primarily a result of quantum mechanics and diffuse scattering and decreases with the reduction in temperature [13,29,32,33], which means that the electrons in the inversion layer are closer to the oxide-silicon interface at lower temperatures. In other words, the electron distribution is closer to ideal as the temperatures decreases. This may explain the reduction in the calculated trap depth and in the $V_{trap}$ − $V_{g}$ slope in Figure 6b.

**Figure 9:**
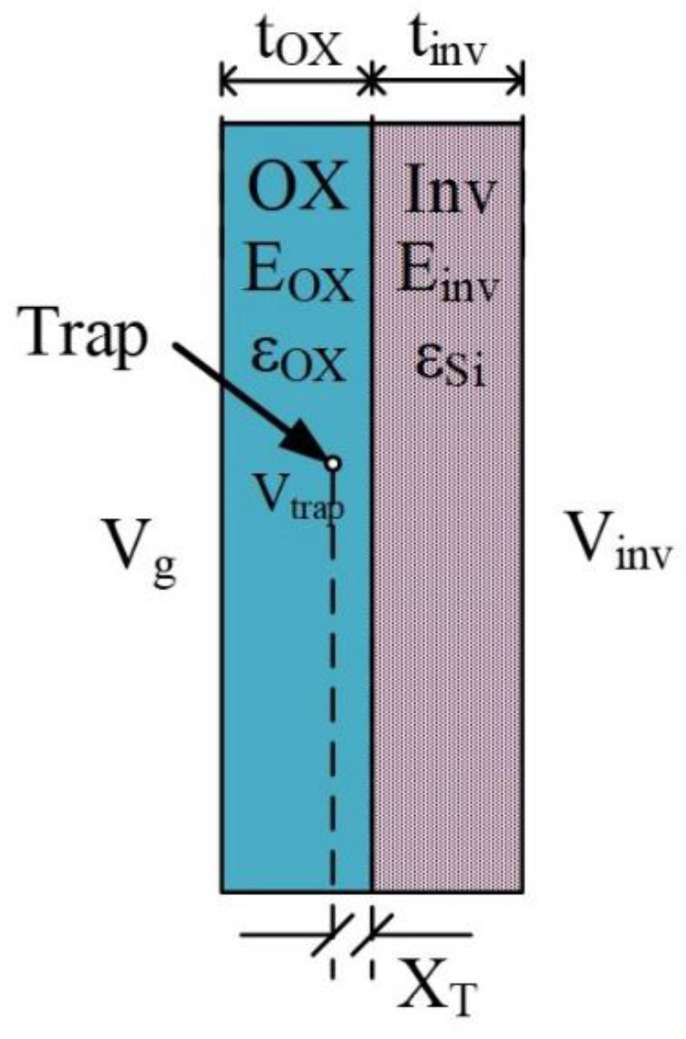
The geometric relationship between the $V_{g}$, $V_{Trap}$, and $X_{T}$ considering the inversion layer thickness.

To explain this issue quantitatively, an improved approach to analyzing the trap depth calculated from the RTN time constants is to be derived. To simplify the calculation, the potential at the inversion layer thickness is assumed to be pinned with respect to $V_{g}$. In addition, the electrical field intensity inside the inversion layer is also assumed to be uniform. These assumptions indicate that the electrons inside the inversion layer are gathered at the inversion layer thickness, i.e., the electron distribution has been ignored. Figure 9 illustrates an improved schematic showing the geometric relationship between $V_{Trap}$ and $X_{T}$ in the inversion region.

From Figure 9, it can be derived:

(9) $$\;E_{OX}=\frac{\;\varepsilon_{Si}}{\;\varepsilon_{OX}}\;E_{inv}$$

(10) $$\;V_{trap}=V_{inv}+E_{inv}\;t_{inv}+E_{OX}\;X_{T}$$

where, $\varepsilon_{Si}$, $\varepsilon_{OX}$, $E_{inv}$, and $t_{inv}$ are the relative permittivity of silicon, the relative permittivity of the gate oxide, the effective electrical field intensity inside the inversion layer, and the inversion layer thickness, repectively. Combining Equations (9) and (10):

(11) $$\;V_{trap}=V_{inv}+E_{inv}\left(\;t_{inv}+\frac{\;\varepsilon_{Si}}{\;\varepsilon_{OX}}\;X_{T}\right)$$

here, $t_{inv}$ is a result of both quantum mechanics and diffuse scattering.

As discussed in [28], the inversion layer thickness due to quantum mechanics at room temperature can be given by:

(12) $$\;t_{inv,QM}=\frac{\beta }{\alpha +E_{inv}{}^{0.7}}$$

where, α = 1 (MV/cm)^0.7^ and β = 1.9 × 10^−7^ cm (MV/cm)^0.7^ [13]. demonstrates that the inversion layer thickness decreases linearly with the reduction in temperature. Thus, in this deduction, it is also assumed that the inversion layer thickness from quantum mechanics decreases linearly with temperature:

(13) $$\;t_{inv,QM}=\frac{\beta }{\alpha +E_{inv}{}^{0.7}}\frac{T}{T_{0}}$$

where, $T_{0}$ is the room temperature.

According to [32], the inversion layer thickness from the diffuse scattering is:

(14) $$\;t_{inv,DS}=\frac{3kT}{2qE_{inv}}$$

So, the total inversion layer thickness becomes:

(15) $$\;t_{inv}=t_{inv,QM}+t_{inv,DS}=\left(\frac{\beta }{\alpha +E_{inv}{}^{0.7}}+\frac{3kT_{0}}{2qE_{inv}}\right)\frac{T}{T_{0}}$$

$E_{inv}$ is given by:

(16) $$\;E_{inv}=\frac{\;V_{gtx}+\alpha \left(\;V_{th}-V_{FB}-\varphi_{s}\right)}{\;2t_{OX}}$$

(17) $$\;V_{gtx}=\eta v_{t}\ln\left[1+\exp\left(\frac{\;V_{g}-V_{th}}{\eta v_{t}}\right)\right]$$

where, $V_{gtx}$ is the auxiliary function of $V_{g}$, α is a fitting parameter, $V_{th}$ is the threshold voltage, $V_{FB}$ is the flat band voltage, $\varphi_{s}$ is the surface potential, η is the sub-threshold swing parameter, and $v_{t}$ is the thermal voltage.

Based on the above discussion, with the increase in temperature, the electrons spread further into the top silicon, which was not considered in the former deduction. Thus, the correction of the electron distribution applied to Equation (11) is:

(18) $$\;V_{trap}=V_{inv}+E_{inv}\left(\;t_{inv}+\frac{\;\varepsilon_{Si}}{\;\varepsilon_{OX}}\;X_{T}\right)+\chi \;V_{g}$$

where, χ is the correction coefficient of the electron distribution and represents the influence of the electron distribution on the $V_{trap}$ − $V_{g}$ slope.

To fit the measured $ln\frac{\tau_{c}}{\tau_{e}}$, both sides of Equation (6) is integrated with respect to $V_{g}$ as:

(19) $$ln\frac{\tau_{c}}{\tau_{e}}=-\frac{q}{kT}\left(V_{Trap}-V_{inv}\right)+Con.$$

where, Con. is the constant of integration. Combining Equations (15), (18) and (19) the fitting to the measured $ln\frac{\tau_{c}}{\tau_{e}}$ can be implemented.

**Figure 10:**
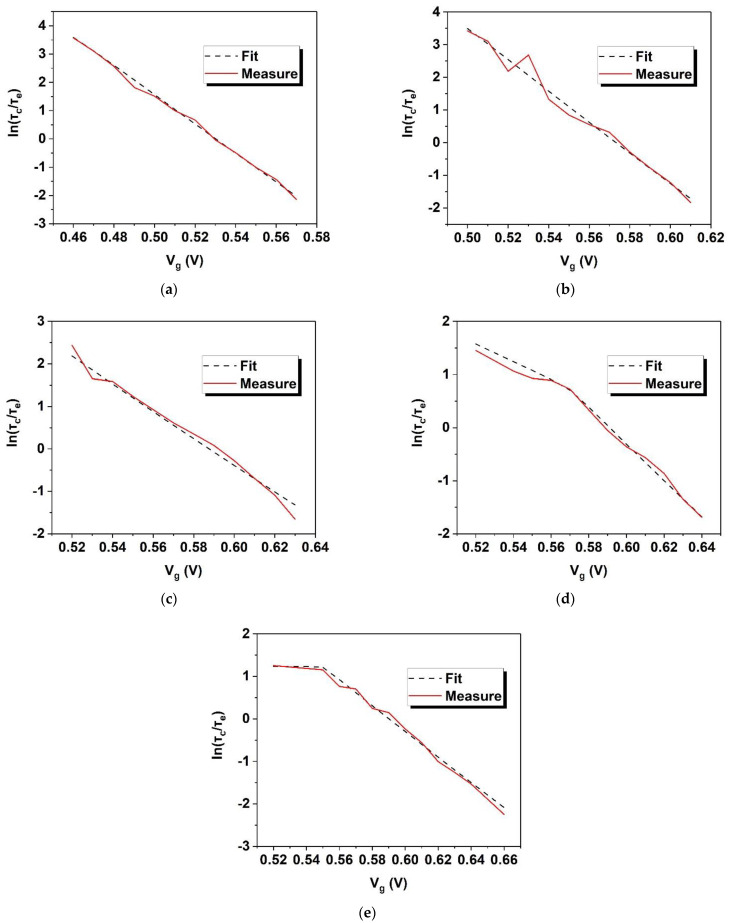
The measured (solid) and fitted (dashed) plots applying the parameters in Table 3 at temperatures of: (**a**) 100 K; (**b**) 77 K; (**c**) 50 K; (**d**) 25 K; and (**e**) 10 K.

Figure 10 shows the fitted plots of $ln\frac{\tau_{c}}{\tau_{e}}$ by $V_{g}$ applying the fitting parameters in Table 3 and using the above deductions. They show good consistency with the measured plots for temperatures from 10 K to 100 K. The absolute errors between measured and fitted $ln\frac{\tau_{c}}{\tau_{e}}$ curves are shown in Table 4. The total maximum error is 0.62478 and the total average error is 0.10574, which are respectively lower by 2.1% and 20% than the conventional linear fitting. Among the fitting parameters, the fitted $X_{T}$ is 0.13 nm and χ decreases from 0.34 to 0 with the reduction in temperature. The trend shown by χ indicates that the electrons are further spread into the top silicon with the rise in temperature, which is consistent with the former assumption.

## 5. Conclusions

This paper proposes an improved approach to analyze the RTN properties of 22-nm FDSOI-based MOSFETs at cryogenic temperatures. The cryogenic measurements of RTN on a 22-nm FDSOI-based DUT have been performed and analyzed using the improved approach, where the quantum mechanical effects and diffuse scattering of electrons inside the top silicon are considered. The basic assumption here is that the $V_{trap}$ by $V_{g}$ slope decreases proportionally with temperature due to the variation of the electron distribution, according to the TCAD simulation results. Applying the improved approach, the calculated and fitted plots of $ln\frac{\tau_{c}}{\tau_{e}}$ by $V_{g}$ is found to be consistent with the measured results earlier. The fitted $X_{T}$ was 0.13 nm, and the decrease of χ proportional with temperature indicates consistency with the aforementioned assumption. This work provides a new method for analyzing RTN in FDSOI MOSFETs at cryogenic temperatures, which plays a significant role in the reliability of cryogenic integration circuits such as the integrated quantum processor. It can also be used to analyze reliability problems caused by RTN in emerging SOI MOSFETs at the cryogenic temperatures.

## Figures and Tables

**Table 1:** The main dimensions of the 22-nm FDSOI MOSFET.

Name	Geometric Size
t_Si_	7 nm
t_BOX_	25 nm
t_OX_	2 nm
L_g_	20 nm
W	160 nm

**Table 2:** The absolute errors between measured and linearly fitted $ln\frac{\tau_{c}}{\tau_{e}}$ curves.

Temperature	Max Error	Average Error
100 K	0.21419	0.06411
77 K	0.63789	0.17187
50 K	0.29193	0.10779
25 K	0.30458	0.14914
10 K	0.54369	0.17107
Total	0.63789	0.13280

**Table 3:** The parameters for fitting the $ln\frac{\tau_{c}}{\tau_{e}}$ plots.

Temperature	$T_{0}$	α	β	η	$\;t_{OX}$	$\;X_{T}$	$\;V_{th}$	$\alpha \left(\;V_{th}-V_{FB}-\varphi_{s}\right)$	χ	Con.
100 K	300 K	1 (MV/cm)^0.7^	1.9 × 10^−7^ cm(MV/cm)^0.7^	1.3	2 nm	0.13 nm	0.46 V	0.05 V	0.34	24.1
77 K							0.49 V	0.05 V	0.23	23.3
50 K							0.49 V	0.08 V	0.08	15.2
25 K							0.57 V	0.12 V	0.036	14.3
10 K							0.55 V	0.15 V	0	7.85

**Table 4:** The absolute errors between the measured and fitted $ln\frac{\tau_{c}}{\tau_{e}}$ curves.

Temperature	Max Error	Average Error
100 K	0.27068	0.06977
77 K	0.62478	0.17199
50 K	0.3329	0.12734
25 K	0.17920	0.07727
10 K	0.16343	0.08233
Total	0.62478	0.10574

## Data Availability

Not applicable.
